# Endoscopic submucosal tunnel implantation of sodium hyaluronate for gastroesophageal reflux disease: a pilot survival porcine study

**DOI:** 10.1055/a-2842-9864

**Published:** 2026-04-20

**Authors:** Pinghua Wen, Yuan Gao, Yi Mou, Xianglei Yuan, Bing Hu

**Affiliations:** 1 34753 Department of Gastroenterology and Hepatology, Digestive Endoscopy Medical Engineering Research Laboratory, West China Hospital of Sichuan University, Chengdu, China

Gastroesophageal reflux disease (GERD) is a chronic gastrointestinal disorder defined by the frequent retrograde flow of gastric contents into the esophagus, causing heartburn and regurgitation 1 . While endoscopic therapy is less invasive than surgery, current methods often cause irreversible anatomical changes with unverified long-term benefits 2 . Specifically, endoscopic submucosal injection has been explored as a potential intervention for GERD, but most materials have failed in practice due to safety issues or lack of efficacy 3 4 . The ideal implantable material for GERD needs to balance multiple properties: good biocompatibility and safety, sufficient mechanical strength, and suitability for endoscopic delivery.

**Fig. 1:**
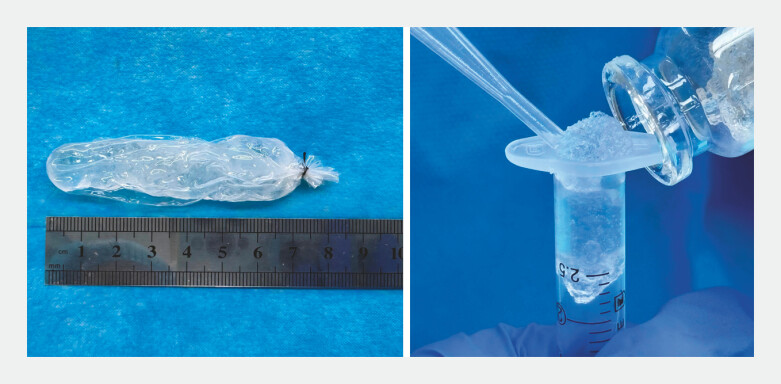
Biocompatible implants (the material was a chemically equivalent precursor to a commercial sodium hyaluronate gel (recognized by CE and NMPA), wrapped with a degradable latex film).

**Fig. 2:**
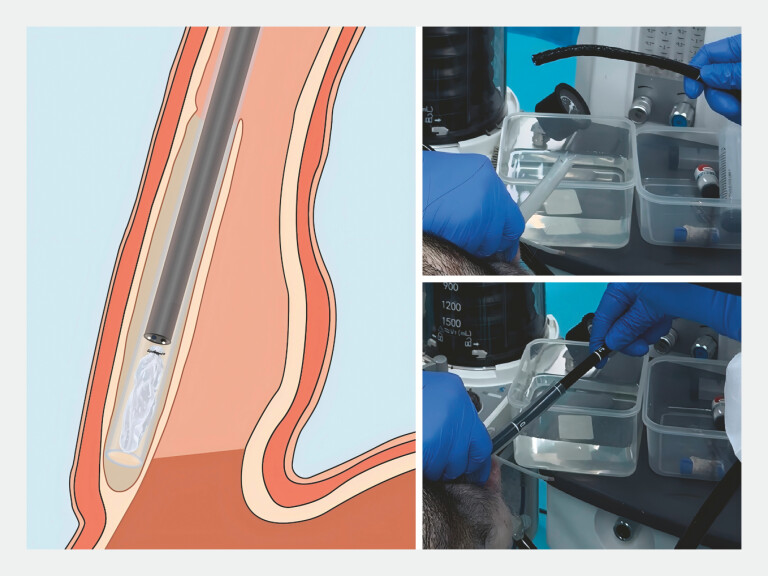
An implant device and a technical diagram: insert the implant into the overtube and use gastroscopy to push it into the submucosal tunnel of the esophagus.

**Fig. 3:**
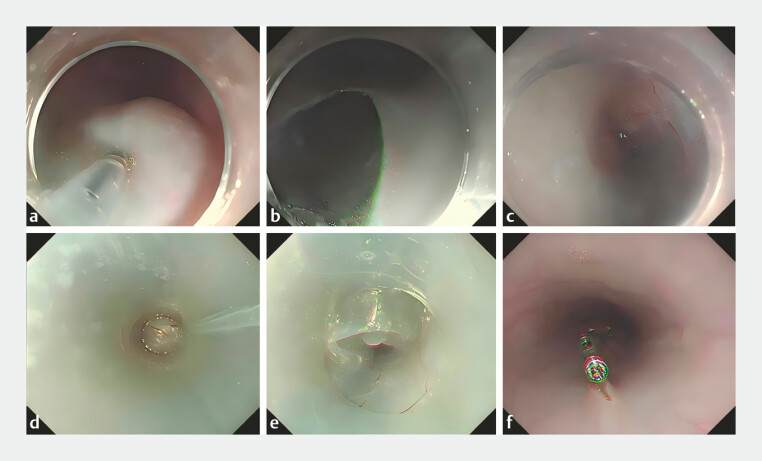
Procedural steps of endoscopic submucosal tunnel implantation. **a** Elevate the mucosa by the submucosal injection of saline. **b** Make a mucosal incision as the tunnel entrance. **c** Dissect the submucosa to establish the tunnel. **d** Advance the gastroscope and overtube into the tunnel. **e** Advance the implant into the tunnel using the gastroscope, and withdraw the overtube carefully. **f** Close tunnel entrance with titanium clips.

**Fig. 4:**
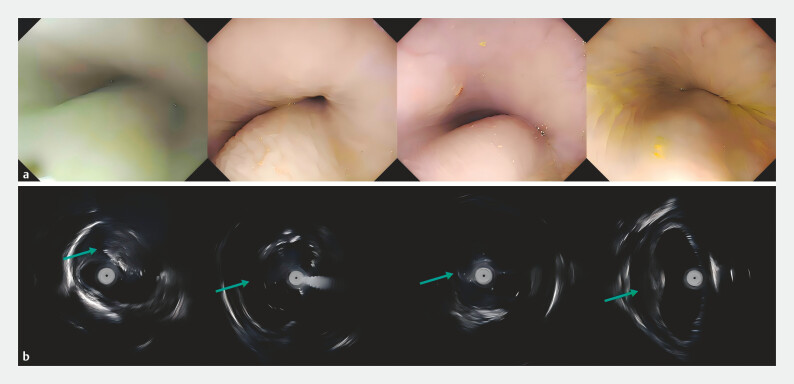
Endoscopic and endosonographic follow-ups. **a** Endoscopic views showing mucosal elevation immediately post-procedure and at 1 week, 2 weeks, and 1 month thereafter. **b** Corresponding endoscopic ultrasonography images demonstrating the position of the implant within the submucosal tunnel at the same time points. (The green arrow indicates the submucosal implant.)

To address these challenges, we developed a novel technique involving endoscopic creation of a submucosal tunnel followed by implantation of a biocompatible sodium hyaluronate strip ( Fig. 1 ). Considering the limited channel size of the endoscope, we assembled the endoscope with an overtube to construct a delivery device ( Fig. 2 ). This approach aims to elevate the mucosa at the gastroesophageal junction, creating a localized, reversible luminal narrowing to reduce reflux. In the porcine model, a submucosal tunnel was established approximately 50 cm from the incisors. A customized overtube was used to position and deliver an 8 cm × 1 cm sodium hyaluronate implant within the tunnel. Finally, the tunnel entrance was closed with titanium clips ( Fig. 3 ). Post-procedure endoscopy confirmed significant mucosal elevation ( Video 1 ). Follow-up evaluations demonstrated the animal's good clinical condition without rejection. Serial endoscopic and endosonographic surveillance confirmed stable implant positioning and sustained mucosal elevation more than 1 month ( Fig. 4 ).

Endoscopic submucosal tunnel implantation of biocompatible sodium hyaluronate in the porcine model.Video 1

Our study proposes an endoscopic strategy characterized by feasibility, potential reversibility, mechanical support, safety, and biodegradability through submucosal tunnel implantation of sodium hyaluronate, indicating its promise for treating GERD. Future studies are required to systematically evaluate its efficacy and long-term safety.

Endoscopy_UCTN_Code_TTT_1AO_2AJ
